# Low-molecular-weight heparin reduces hyperoxia-augmented ventilator-induced lung injury via serine/threonine kinase-protein kinase B

**DOI:** 10.1186/1465-9921-12-90

**Published:** 2011-07-05

**Authors:** Li-Fu Li, Cheng-Ta Yang, Chung-Chi Huang, Yung-Yang Liu, Kuo-Chin Kao, Horng-Chyuan Lin

**Affiliations:** 1 Department of Medicine, Division of Pulmonary and Critical Care Medicine, Chang Gung Memorial Hospital, Kweishan, Taoyuan, Taiwan; 2 Chang Gung University, Kweishan, Taoyuan, Taiwan; 3 Department of Respiratory Therapy, Chang Gung Memorial Hospital, Kweishan, Taoyuan, Taiwan; 4 Chest Department, Veterans General Hospital, Taipei, Taiwan; 5 National Yang-Ming University, School of Medicine, Taipei, Taiwan

## Abstract

**Background:**

High-tidal-volume mechanical ventilation and hyperoxia used in patients with acute lung injury (ALI) can induce the release of cytokines, including high-mobility group box-1 (HMGB1), oxygen radicals, neutrophil infiltration, and the disruption of epithelial and endothelial barriers. Hyperoxia has been shown to increase ventilator-induced lung injury, but the mechanisms regulating interaction between high tidal volume and hyperoxia are unclear. We hypothesized that subcutaneous injections of enoxaparin would decrease the effects of hyperoxia on high-tidal-volume ventilation-induced HMGB1 production and neutrophil infiltration via the serine/threonine kinase/protein kinase B (Akt) pathway.

**Methods:**

Male C57BL/6, either wild type or Akt^+/-^, aged between 6 and 8 weeks, weighing between 20 and 25 g, were exposed to high-tidal-volume (30 ml/kg) mechanical ventilation with room air or hyperoxia for 2 to 8 hours with or without 4 mg/kg enoxaparin administration. Nonventilated mice served as a control group. Evan blue dye, lung wet-to-dry weight ratio, free radicals, myeloperoxidase, Western blot of Akt, and gene expression of HMGB1 were measured. The expression of HMGB1 was studied by immunohistochemistry.

**Results:**

High-tidal-volume ventilation using hyperoxia induced microvascular permeability, Akt activation, HMGB1 mRNA expression, neutrophil infiltration, oxygen radicals, HMGB1 production, and positive staining of Akt in bronchial epithelium. Hyperoxia-induced augmentation of ventilator-induced lung injury was attenuated with Akt deficient mice and pharmacological inhibition of Akt activity by enoxaparin.

**Conclusion:**

These data suggest that enoxaparin attenuates hyperoxia-augmented high-tidal-volume ventilation-induced neutrophil influx and HMGB1 production through inhibition of the Akt pathway. Understanding the protective mechanism of enoxaparin related with the reduction of HMGB1 may help further knowledge of the effects of mechanical forces in the lung and development of possible therapeutic strategies involved in acute lung injury.

## Introduction

Acute respiratory distress syndrome (ARDS)/acute lung injury (ALI) is characterized by noncardiogenic pulmonary edema, the release of cytokines, and the influx of neutrophils [1]. The management of ARDS often requires the use of mechanical ventilation with high levels of oxygen to maintain adequate blood oxygenation. Concurrent hyperoxia and high tidal volume (V_T_) mechanical ventilation may interact to promote worsening acute lung injury and lead to the production of inflammatory cytokines, including macrophage inflammatory protein-2 (MIP-2), tumor necrosis factor-α (TNF-α), plasminogen activator inhibitor-1 (PAI-1), and high-mobility group box-1 (HMGB1) [2-5].

In previous studies of ventilator-induced lung injury (VILI) in mice and rats, hyperoxia was involved in the pathogenesis of alveolar inflammation and was characterized by lung edema, sequestration of neutrophils, destruction of type II alveolar epithelial cells, prominent intraalveolar deposition of fibrin and cell debris, the classical feature of hyaline membrane disease [3,6]. The major oxidants in airways are reactive oxygen species, including superoxide, hydrogen peroxide, and hydroxyl radical. Reactive oxygen species may overwhelm the protection of antioxidants in acute lung injury [7,8].

HMGB1 is expressed in various cells, including macrophages/monocytes, endothelial cells, neutrophils, epithelial cells, dendritic cells, and smooth muscle cells [9]. Because HMGB1 is present in most cell types and can be passively released from necrotic cells, it may be released into bronchoalveolar lavage fluid from injured epithelial cells in VILI [10,11]. HMGB1 can stimulate the release of cytokines, and conversely, cytokines can control the further release of HMGB1 to the extracellular space. During endotoxemia HMGB1 rises in the circulation starting at 8 hours, increasing until 16 hours, and thereafter remains at a high level until 36 h [12]. Although it is a late mediator of lung injury, HMGB1 might express its toxicity in a short period of time in VILI [10], even in patients without previous lung injury [13]. High-tidal-volume mechanical ventilation can also lead to increase of HMGB1 production, recruitment of neutrophils, and the development of acute lung injury involving activation of phosphoinositide 3-OH kinase (PI 3-K) and serine/threonine kinase/protein kinase B (Akt) in an isolated mouse model of endotoxemia [14].

Enoxaparin, one of the low-molecular-weight heparins (LMWHs), is safer and easier to administer than unfractionated heparin, a naturally occurring glycosaminoglycan with both anticoagulant and anti-inflammatory activities [15]. Low dose of LMWH was found in a murine acute lung injury model to have protective effects due to its effects on neutrophil emigration, edema formation, and PAI-1 production [16]. No significant side effects of airway bleeding were found. Dixon and coworkers showed that nebulized heparin was associated with fewer days of mechanical ventilation in critically ill patients but the mechanism was unclear [17]. In this high-tidal-volume ventilation-induced acute lung injury model in mice with hyperoxia, we explored the effects of low dose of LMWH on high-tidal-volume ventilation-induced neutrophil infiltration and correlation of acute lung injury to the production of HMGB1 and the activation of Akt signaling using animals deficient in Akt. We hypothesized that subcutaneous injections of enoxaparin would decrease neutrophil influx, lung edema, and HMGB1 production in mice exposed to high-tidal-volume ventilation with hyperoxia via Akt pathway.

## Materials and methods

### Experimental animals

Male C57BL/6, either wild type or Akt^+/- ^on a C57BL/6 background, weighing between 20 and 25 g, aged between 6 and 8 weeks, were obtained from Jackson Laboratories (Bar Harbor, ME) and National Laboratory Animal Center (Taipei, Taiwan) as previously described [18]. Briefly, Heterozygotes are used because homozygotes exhibit lower fertility and female homozygotes do not nurse well; up to 50% perinatal mortality can occur. Mice that are heterozygous for the targeted mutation are viable and do not display any gross behavioral abnormalities. The construct Akt containing disrupted exons 4 through 7 and the 5'end of exon 8 is electroporated into 129P2Ola/Hsd derived E14 embryonic stem cells. Chimeras are generated by injecting these ES cells into C57BL/6 (B6) blastocysts. The resulting chimeric male animals are crossed to C57BL/6 mice, and then backcrossed to the same for 10 generations. Heterozygotes are intercrossed to generate homozygous mutant mice. In our murine model of ALI, we used male mice to decrease the effects of estrous cycles found in female mice [6]. The lower expressions of the Akt protein in Akt^+/- ^mice were confirmed using Western blot analysis. The study was performed in strict accordance with the recommendations in the Guide for the Care and Use of Laboratory Animals of the National Institutes of Health. The protocol was approved by the Institutional Animal Care and Use Committee of Chang Gung Memorial Hospital (Permit Number: 2008090102). All surgery was performed under ketamine and xylazine anesthesia, and all efforts were made to minimize suffering.

### Ventilator Protocol

We used our established mouse model of VILI, as previously described [6]. In brief, tracheostomy was performed under general anesthesia with intraperitoneal ketamine (90 mg/kg) and xylazine (10 mg/kg) followed by ketamine (0.1 mg/g/h) and xylazine (0.01 mg/g/h) at a rate of 0.09 ml/10 g/h by a continuous intraperitoneal infusion. The mice were placed in a supine position on a heating blanket and then attached to a Harvard apparatus ventilator, model 55-7058 (Harvard Apparatus, Holliston, MA), set to deliver 30 ml/kg at a rate of 65 breaths per minute, for 2 to 8 hours while breathing room air or hyperoxia (> 95% oxygen) with zero end-expiratory pressure. Oxygen was fed into the inspiratory port of the ventilator when needed. Spontaneously breathing animals were exposed to hyperoxia in an enclosed chamber as previously described [2]. Two hours of mechanical ventilation for RT-PCR and Western blot and 8 hours for HMGB1 production, free radicals, and immunohistochemical analysis were used based on our time-course and previous studies [12]. The tidal volume delivered by the ventilator was checked by fluid displacement from an inverted calibration cylinder. Continuous monitoring of end-tidal CO_2 _by a microcapnograph (Columbus Instruments, Columbus, OH) was performed, and respiratory frequencies of 65 breaths per minute for 30 ml/kg were chosen in the experiment, with end-tidal CO_2 _at 30 to 40 mm Hg. Airway peak inspiratory pressure was measured with a pressure-transducer amplifier (Gould Instrument Systems, Valley View, OH) connected to the tubing at the proximal end of the tracheostomy. Mean arterial pressure was monitored every hour during mechanical ventilation by using the same pressure-transducer amplifier connected to a 0.61-mm outer diameter (0.28-mm inner diameter) polyethylene catheter ending in the common carotid artery. At the end of the study period, heparinized blood was taken from the arterial line for analysis of arterial blood gas, and the mice were sacrificed. Control, nonventilated mice were anesthetized and sacrificed immediately. The experimental design and numbers of animals per group is summarized in Table 1.

**Table 1:** Experimental design and numbers of animals per group

	Control + RA (WT)	Control + O_2 _(WT)	V_T _30 ml/kg + RA (WT)	V_T _30 ml/kg + O_2 _(WT)	V_T _30 ml/kg + O_2 _with enoxaparin (WT)	V_T _30 ml/kg + O_2 _(Akt^+/-^)
HMGB1, cell isolation (8 hours of ventilation)	5	5	5	5	5	5
Phospho-Akt, HMGB1 mRNA (2, 4, 8 hours of ventilation)	5	5	5 × 3	5 × 3	5	5
MPO, lung edema (8 hours of ventilation)	5	5	5	5	5	5
EBD assay (8 hours of ventilation)	5	5	5	5	5	5
MDA/GSH, IHC (8 hours of ventilation)	5	5	5	5	5	5

Akt = serine/threonine kinase/protein kinase B; Akt^+/- ^= Akt deficient mice; Control = spontaneously breathing, nonventilated mice; EBD = Evans blue dye; GSH = total glutathione; HMGB1 = high-mobility group box-1; IHC = immunohistochemical stain; MDA = malondialdehyde; MPO = myeloperoxidase; O_2 _= mice with hyperoxia; RA = mice with room air; V_T _= tidal volume; WT: wild type.

### Enoxaparin administration

Low dose of enoxaparin (Sigma, St. Louis, MO), 4 mg/kg, was given subcutaneously 30 minutes before ventilation, based on our previous *in vivo *study that showed 4 mg/kg enoxaparin inhibited blood coagulation and lung injury without significant bleeding tendency [16].

### Isolation of intrapulmonary neutrophils

The lungs were minced finely, and incubated in RPMI 1640 with 10 mM HEPES, 20 mM L-glutamine, 10% fetal cuff serum, 1% penicillin-streptomycin, 20 U/ml of collagenase, and 1 μg/ml of type I DNase (Sigma, St. Louis, MO) [19]. After incubation for 60 minutes at 37°C on a rotary agitator, any remaining intact tissue was disrupted by passage through a 21-gauge needle. Tissue fragments and the majority of dead cells were removed by rapid filtration through a filtration tube (Sigma, St. Louis, MO), and cells collected by centrifugation. Intrapulmonary neutrophils were isolated using the anti-Ly-6G microbead neutrophil isolation kit with high purities of more than 95% of neutrophils (Miltenyi Biotec Inc., Auburn, CA). The cell pellet was flash frozen for the Western blot analysis.

### Evans Blue Dye Analysis

Extravasation of Evans blue dye (Sigma Chemical, St. Louis, MO) into the interstitium was used as a quantitative measure of changes of microvascular permeability in acute lung injury [6]. Thirty minutes before end of mechanical ventilation, 30 mg/kg of Evans blue dye was injected through the internal jugular vein. At the time of death after 8 hours of mechanical ventilation, the lungs were perfused free of blood with 1 ml of 0.9% normal saline via the right ventricle and removed *en bloc*. Evans blue was extracted from lung tissue after homogenization for 2 minutes in 5 ml of formamide (Sigma Chemical, St. Louis, MO) and incubated at 37°C overnight. The supernatant was separated by centrifugation at 5,000 *g *for 30 minutes, and the amount was recorded. Evans blue in the plasma and lung tissue was quantitated by dual-wave-length spectrophotometric analysis at 620 and 740 nm. The method corrects the specimen's absorbance at 620 nm for the absorbance of contaminating heme pigments, by using the following formula: corrected absorbance at 620 = actual absorbance at 620 nm-[1.426 (absorbance at 740) + 0.03]. We calculated the Evans blue dye amount extracted from lung tissue and divided the amount by the weight of the lung tissue.

### Analysis of lung water

Lungs were removed *en bloc*, and large airways were removed. Both lungs were weighed and then dried in an oven at 80°C for 48 hours. If no changes were found in the dry lung weight at 24 and 48 hours, the weight at 48 hours was used. Lung wet-to-dry weight ratio was used as an index of pulmonary edema formation [16].

### Myeloperoxidase Assay

The lungs (0.12 to 0.17 g) were homogenized in 5 ml of phosphate buffer (20 mM, pH 7.4). One milliliter of the homogenate was centrifuged at 10,000 *g *for 10 minutes at 4°C. The resulting pellet was resuspended in 1 ml of phosphate buffer (50 mM, pH 6.0) containing 0.5% hexadecyltrimethylammonium bromide. The suspension was then subjected to three cycles of freezing (on dry ice) and thawing (at room temperature), after which it was sonicated for 40 seconds and centrifuged again at 10,000 *g *for 5 minutes at 4°C. The supernatant was assayed for MPO activity by measuring the hydrogen peroxide (H_2_O_2_)-dependent oxidation of 3, 3', 5, 5'-tetramethylbenzidine (TMB). In its oxidized form, TMB has a blue color, which was measured spectrophotometrically at 650 nm. The reaction mixture for analysis consisted of a 25-μl tissue sample, 25 μl of TMB (final concentration 0.16 mM) dissolved in dimethylsulfoxide, and 200 μl of H_2_O_2 _(final concentration 0.30 mM) dissolved in phosphate buffer (0.08 M, pH 5.4). The reaction mixture was incubated for 3 minutes at 37°C and the reaction was stopped by adding 1 ml of sodium acetate (0.2 M, pH 3.0), after which absorbance at 650 nm was measured. The absorbance (A650) was reported as optical density (OD)/g of wet lung weight [6].

### Immunoblot analysis

The lungs were homogenized in 3 ml of lysis buffer (20 mM HEPES pH 7.4, 1% Triton X-100, 10% glycerol, 2 mM ethylene glycol-bis (β-aminoethyl ether)-N, N, N', N'-tetraacetic acid, 50 μM β-glycerophosphate, 1 mM sodium orthovanadate, 1 mM dithiotreitol, 400 μM aprotinin, and 400 μM phenylmethylsulfonyl fluoride), transferred to eppendorff tubes and placed on ice for 15 minutes. Tubes were centrifuged at 14,000 rpm for 10 minutes at 4°C and supernatant was flash frozen. Crude cell lysates were matched for protein concentration, resolved on a 10% bis-acrylamide gel, and electrotransferred to Immobilon-P membranes (Millipore Corp., Bedford, MA). For assay of Akt phosphorylation and Akt total protein expression, Western blot analyses were performed with antibodies of phospho-Akt and Akt (New England BioLabs, Beverly, MA). Blots were developed by enhanced chemiluminescence (NEN Life Science Products, Boston, MA).

### Reverse transcription-polymerase chain reaction

For isolating total RNA, the lung tissues were homogenized in TRIzol reagents (Invitrogen Corporation, Carlsbad, CA) according to the manufacturer's instructions. Total RNA (1 μg) was reverse transcribed by using a GeneAmp PCR system 9600 (PerkinElmer, Life Sciences, Inc., Boston, MA), as previously described [16]. The following primers were used for PCR: HMGB1, forward primer 5'-TGGCAAAGGCTGACAAGGCTC-3' and reverse primer 5'-GGATGCTCGCCTTTGATTTTGG-3' and GAPDH as internal control, forward primer 5'-AATGCATCCTGCA CCACCAA-3' and reverse primer 5'-GTAGCCATATTCATTGTCATA-3' (Integrated DNA Technologies, Inc., Coralville, IA)[20].

### Measurement of malondialdehyde and total glutathione

The lungs were homogenized in phosphate buffered saline containing butylated hydroxytoluene for MDA and metaphosphoric acid for total glutathione. The malondialdehyde and total glutathione in the protein extracts were measured using the Oxiselect TBARS assay kit containing thiobarbituric acid reactive substances and Oxiselect total glutathione assay kit containing glutathione reductase (Cell Biolabs, San Diego, CA). Each sample was run in duplicate and expressed as μmole/g protein for MDA and μg/mg protein for total glutathione according to the manufacturer's instructions.

### Measurement of HMGB1

At the end of the study period, the lungs were lavaged via tracheostomy with 20-gauge angiocatheter (sham instillation) 3 times with 0.6 ml of 0.9% normal saline. The effluents were pooled and centrifuged at 2,000 rpm for 10 minutes. Supernatants were frozen at -80°C for further analysis of the cytokine. HMGB1 with a lower detection limit of 1 ng/ml was measured in bronchoalveolar lavage fluid using a commercially available immunoassay kit containing primary polyclonal anti-mouse antibody that was cross-reactive with rat and mouse HMGB1 (Shino-Test corporation, Kanagawa, Japan). Each sample was run in duplicate according to the manufacturer's instructions.

### Immunohistochemistry

The lungs were paraffin embedded, sliced at 4 μm, deparaffinized, antigen unmasked in 10 mM sodium citrate (pH 6.0), incubated with rabbit phospho-Akt primary antibody (1:100; New England BioLabs, Beverly, MA), and biotinylated goat anti-rabbit secondary antibody (1:100) according to the manufacturer's instruction for an immunohistochemical kit (Santa Cruz Biotechnology, Santa Cruz, CA) [6].

### Statistical Evaluation

The Western blots and HMGB1 mRNA were quantitated using a National Institutes of Health (NIH) image analyzer, ImageJ 1.27z (National Institute of Health, Bethesda, MD, USA) and presented as arbitrary units. Values were expressed as the mean ± SD for at least 5 experiments. The data of Evans blue dye (EBD) assay, lung wet-to-dry weight ratio, HMGB1, myeloperoxidase (MPO), immunohistochemical assay, MDA, and total glutathione were conducted by using Statview 5.0 (Abascus Concepts Inc. Cary, NC, USA; SAS Institute, Inc.). All results of Western blots and HMGB1 mRNA were normalized to control, nonventilated mice with room air. ANOVA was used to assess the statistical significance of the differences, followed by multiple comparisons with a Scheffe's test, and a *P *value < 0.05 was considered statistically significant. Regression coefficients were calculated using the simple regression test in Statview.

## Results

### Physiologic data

No statistical difference was found in pH, PaO_2_, PaCO_2_, mean arterial pressure, and peak inspiratory pressure at the beginning versus the end of 8 hours of mechanical ventilation (Table 2). The normovolemic statuses of mice were maintained by monitoring mean artery pressure.

**Table 2:** Physiologic conditions at the beginning and end of ventilation

	Nonventilated Room air	Nonventilated Hyperoxia	V_T _30 ml/kg Room air	V_T _30 ml/kg Hyperoxia
PH	7.40 ± 0.05	7.37 ± 0.01	7.35 ± 0.04	7.34 ± 0.02
PaO2 (mmHg)	98.6 ± 0.3	423.5 ± 4.3	86.9 ± 0.7	399.7 ± 3.6
PaCO2 (mmHg)	39.1 ± 0.2	39.6 ± 0.7	37.3 ± 1.2	43.4 ± 1.5
MAP (mmHg)				
Start	85.5 ± 1.2	85.9 ± 1.9	84.8 ± 1.5	84.5 ± 2.4
End	85.1 ± 0.6	84.9 ± 0.8	77.5 ± 3.0	77.3 ± 2.3
PIP (mmHg)				
Start			24.3 ± 1.2	24.9 ± 1.3
End			27.7 ± 1.0	28.2 ± 1.3

Arterial blood gases and mean arterial pressure were obtained from nonventilated mice and mice ventilated at a tidal volume of 30 ml/kg for 8 hours (n = 10 per group). MAP = mean arterial pressure; PIP = peak inspiratory pressure; V_T _= tidal volume. The physiological data of control groups were similar during the experiment and were used as the beginning data of mechanical ventilation. Because no differences were observed among hyperoxia with enoxaparin, without enoxaparin, and Akt deficient groups, the data were combined.

### Inhibition of the effects of hyperoxia on lung stretch-induced microvascular leak, oxygen radicals, neutrophil sequestration, and HMGB1 production with enoxaparin

**Figure 1:**
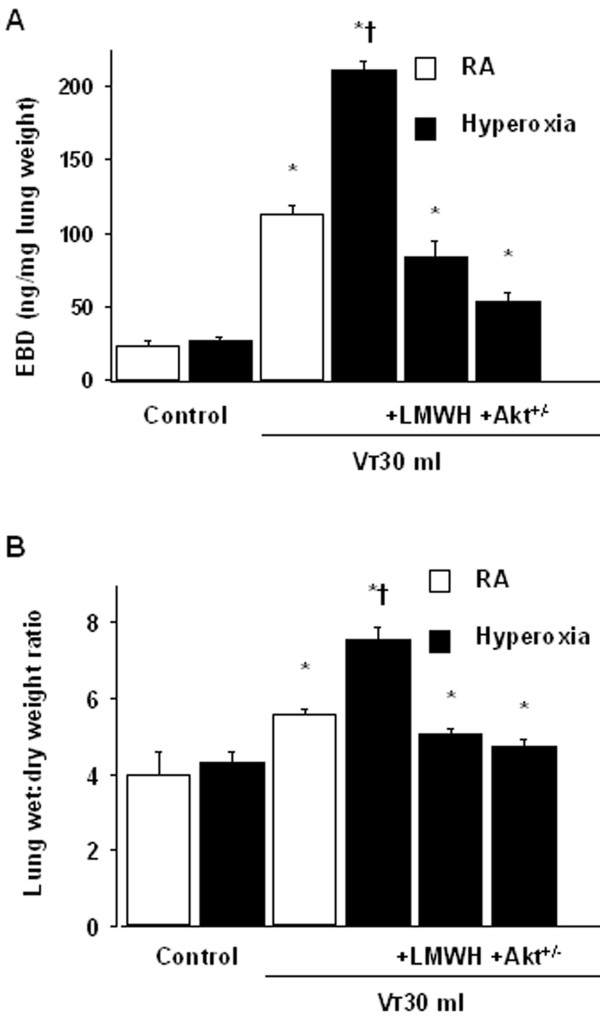
**Enoxaparin and Akt deficient mice reduced hyperoxia-augmented lung stretch-induced microvascular leak and lung edema**. Evans blue dye (EBD) analysis of lung tissue (A) and lung water measured by lung wet-to-dry-weight ratio (B) were from control, nonventilated mice and mice ventilated at tidal volume (V_T_) 30 ml/kg (V_T _30 ml) for 8 hours with room air or hyperoxia. Enoxaparin, 4 mg/kg, was given subcutaneously 30 minutes before ventilation (n = 5 per group). * *P *< 0.05 versus control, nonventilated mice; **†***P *< 0.05 versus all other groups. Akt^+/- ^= serine/threonine kinase/protein kinase (Akt) deficient mice; LMWH = enoxaparin; RA = mice with room air.

**Figure 2:**
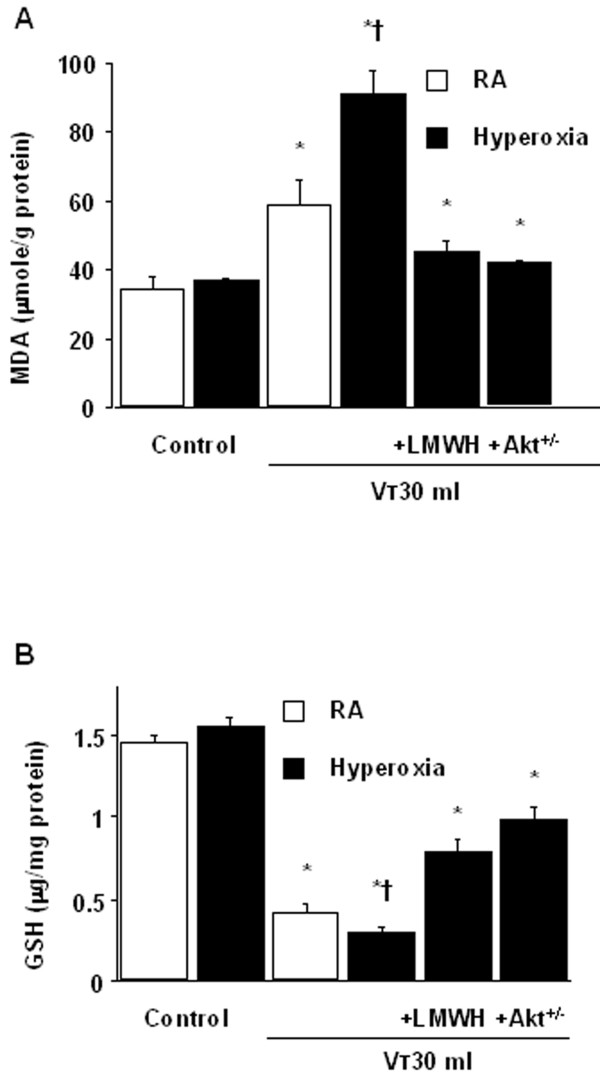
**Enoxaparin and Akt deficient mice reduced the effects of hyperoxia on lung stretch-induced oxidant stress**. Malondialdehyde (MDA) assay (A) and total glutathione (GSH) assay (B) of lung tissue were from control, nonventilated mice and mice ventilated at V_T _30 ml/kg for 8 hours with room air or hyperoxia. Enoxaparin, 4 mg/kg, was given subcutaneously 30 minutes before ventilation (n = 5 per group). * *P *< 0.05 versus control, nonventilated mice; **†***P *< 0.05 versus all other groups. Akt^+/- ^= Akt deficient mice; LMWH = enoxaparin; RA = mice with room air.

**Figure 3:**
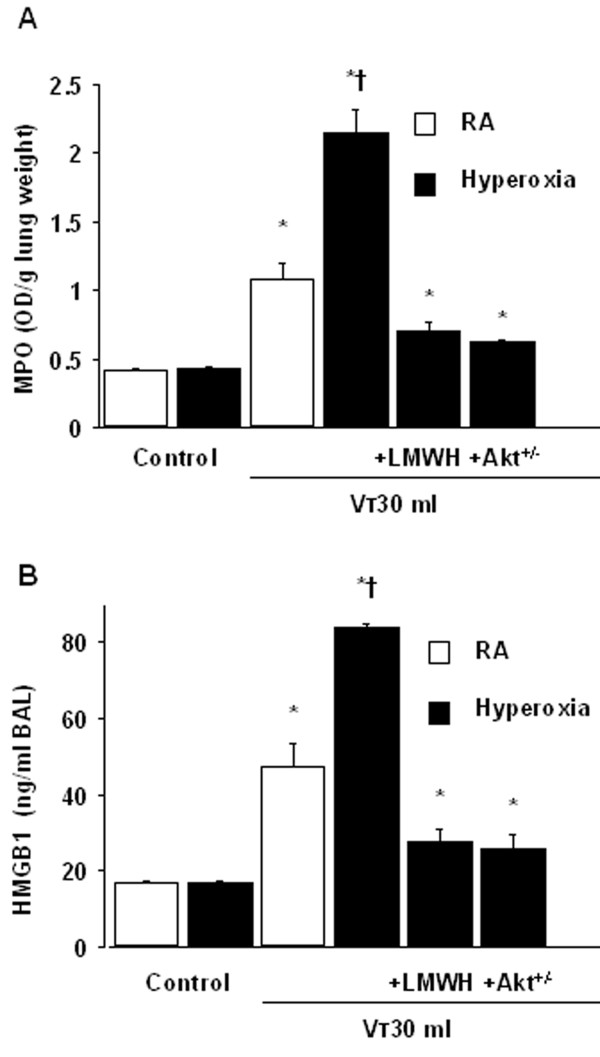
**Enoxaparin and Akt deficient mice reduced hyperoxia-augmented lung stretch-induced neutrophil sequestration and HMGB1 production**. Myeloperoxidase (MPO) assay of lung tissue (A) and high-mobility group box-1 (HMGB1) production in bronchoalveolar lavage (BAL) fluid (B) were from control, nonventilated mice and mice ventilated at V_T _30 ml/kg for 8 hours with room air or hyperoxia. Enoxaparin, 4 mg/kg, was given subcutaneously 30 minutes before ventilation (n = 5 per group). * *P *< 0.05 versus control, nonventilated mice; **†***P *< 0.05 versus all other groups. Akt^+/- ^= Akt deficient mice; LMWH = enoxaparin; RA = mice with room air.

To determine the effects of hyperoxia on changes of microvascular permeability and lung water in VILI, we measured lung EBD and wet-to-dry weight ratio. The levels of lung EBD and wet-to-dry weight ratio were significantly increased in mice receiving V_T _30 ml/kg mechanical ventilation with hyperoxia compared with those of control, nonventilated mice and mice ventilated with room air at a V_T _of 30 ml/kg (Figure 1). To measure the level of oxidant stress involved in hyperoxia-induced ALI, we measured MDA and total glutathione levels from lung tissues. Increased production of MDA but reduced production of total glutathione was found in mice ventilated at V_T _30 ml/kg with hyperoxia compared with those of control, nonventilated mice and mice ventilated with room air at a V_T _of 30 ml/kg (Figure 2). To determine the effects of hyperoxia on neutrophils, a potential source of reactive oxygen species marginated in the vasculature, lung parenchyma, and in the alveoli, and to determine the inflammatory cytokines for neutrophils, we measured MPO activity and HMGB1 protein production. The MPO levels and HMGB1 protein production in mice ventilated with hyperoxia at V_T _30 ml/kg were significantly elevated compared with those of control, nonventilated mice and mice ventilated with room air at a V_T _of 30 ml/kg (Figures 3). The increases in microvascular leak, lung edema, MPO levels, and HMGB1 production in mice receiving V_T _30 ml/kg mechanical ventilation with hyperoxia were significantly reduced after pharmacological inhibition with enoxaparin. Reduced production of MDA but increased production of total glutathione was found after pharmacological inhibition with enoxaparin.

### Inhibition of the effects of hyperoxia on lung stretch-induced HMGB1 mRNA expression with enoxaparin

**Figure 4:**
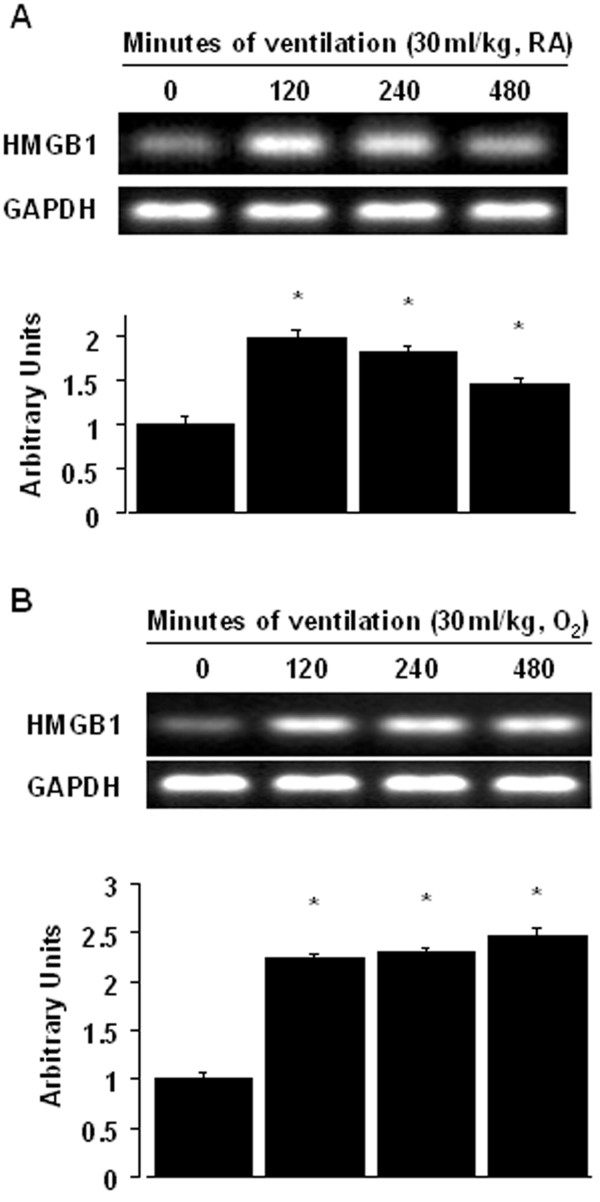
**High tidal volume ventilation caused a time-dependent increase on HMGB1 mRNA expression**. Reverse transcription-polymerase chain reaction (RT-PCR) assay was performed for HMGB1 mRNA (A and B, top panel), glyceraldehydes-phosphate dehydrogenase (GAPDH) mRNA (A and B, middle panel), and arbitrary units (A and B, bottom panel) from control, nonventilated mice and mice ventilated with V_T _30 ml/kg breathing room air or hyperoxia at indicated time periods (n = 5 per group). Arbitrary units were expressed as the ratio of HMGB1 mRNA to GAPDH. * *P *< 0.05 versus control, nonventilated mice. O_2 _= mice with hyperoxia; RA = mice with room air.

**Figure 5:**
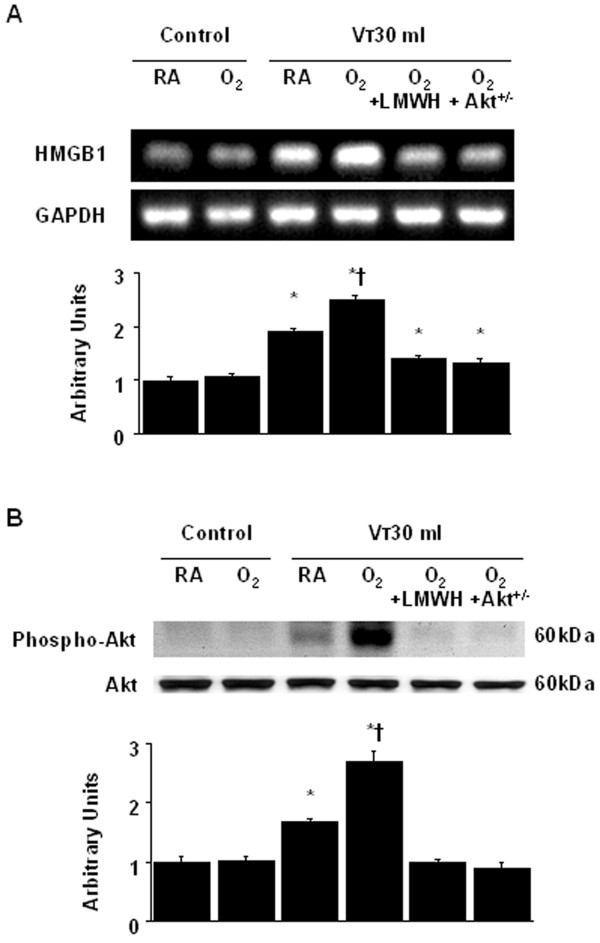
**Enoxaparin and Akt deficient mice reduced hyperoxia-augmented lung stretch-induced HMGB1 mRNA expression and Akt activation**. Wild-type or Akt^+/- ^mice were ventilated at V_T _30 ml/kg for 2 hours with room air or hyperoxia. Enoxaparin, 4 mg/kg, was given subcutaneously 30 minutes before ventilation. (A) RT-PCR assay was performed for HMGB1 mRNA (A, top panel), GAPDH mRNA (A, middle panel), and arbitrary units (A, bottom panel). Arbitrary units were expressed as the ratio of HMGB1 mRNA to GAPDH (n = 5 per group). (B) Western blot was performed using an antibody that recognizes the phosphorylated Akt expression (B, top panel) and an antibody that recognizes total Akt protein expression in lung tissue homogenate (B, middle panel). Arbitrary units were expressed as relative Akt phosphorylation (B, bottom panel) (n = 5 per group). * *P *< 0.05 versus control, nonventilated mice; **†***P *< 0.05 versus all other groups. Akt^+/- ^= Akt deficient mice; LMWH = enoxaparin; O_2 _= mice with hyperoxia; RA = mice with room air.

To determine whether the increased neutrophil influx in mice receiving V_T _30 ml/kg mechanical ventilation was associated with upregulation of chemotactic factors for neutrophils, we measured HMGB1 mRNA expression for 2 to 8 hours of mechanical ventilation while breathing room air or hyperoxia. The HMGB1 mRNA expression was increased at 2 hours of mechanical ventilation with V_T _30 ml/kg and remained elevated after 8 hours of mechanical ventilation compared with those of control, nonventilated mice (Figures 4). The increases in HMGB1 mRNA expression in mice receiving V_T _30 ml/kg mechanical ventilation with hyperoxia were significantly reduced after pharmacological inhibition with enoxaparin (Figure 5A). The reduction between V_T _30 ml/kg mechanical ventilation using hyperoxia with and without enoxaparin was more than those between V_T _30 ml/kg mechanical ventilation with and without hyperoxia. This suggested that pharmacological inhibition with enoxaparin reduced both effects of mechanical ventilation and hyperoxia.

### Inhibition of the effects of hyperoxia on lung stretch-induced Akt activation with enoxaparin

**Figure 6:**
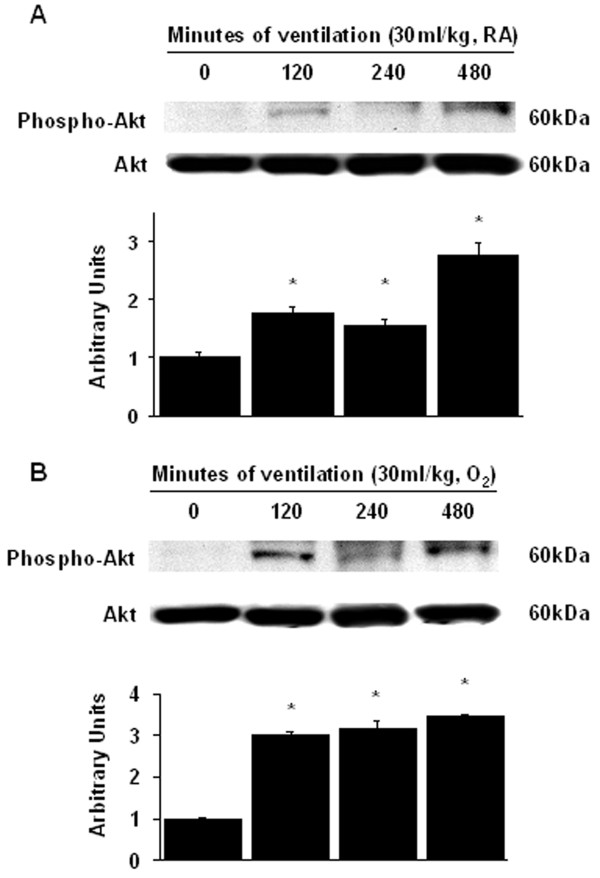
**High tidal volume ventilation caused a time-dependent increase on Akt activation**. Western blot was performed using an antibody that recognizes the phosphorylated Akt expression (A and B, top panel) and an antibody that recognizes total Akt protein expression in lung tissue homogenate (A and B, middle panel) from control, nonventilated mice, and mice ventilated with V_T _30 ml/kg breathing room air or hyperoxia at indicated time periods. Arbitrary units were expressed as relative Akt phosphorylation (A and B, bottom panel) (n = 5 per group). * *P *< 0.05 versus control, nonventilated mice. O_2 _= mice with hyperoxia; RA = mice with room air.

**Figure 7:**
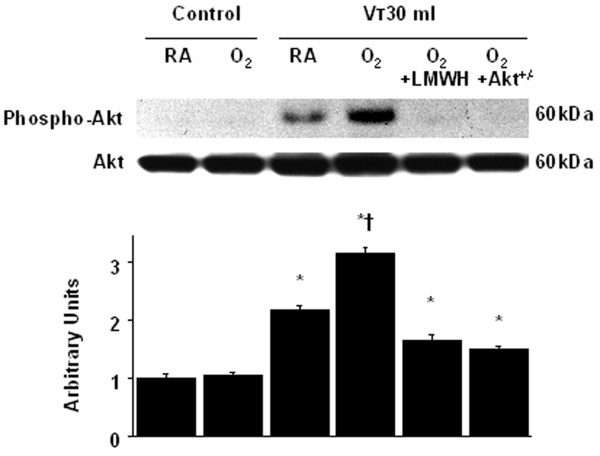
**Enoxaparin and Akt deficient mice reduced hyperoxia-augmented lung stretch-induced Akt activation in neutrophils**. Wild-type or Akt^+/- ^mice were ventilated at V_T _30 ml/kg for 2 hours with room air or hyperoxia. Enoxaparin, 4 mg/kg, was given subcutaneously 30 minutes before ventilation. Western blot from isolated neutrophils of lung tissue was performed using an antibody that recognizes the phosphorylated Akt expression (top panel) and an antibody that recognizes total Akt protein expression in lung tissue (middle panel). Arbitrary units were expressed as relative Akt phosphorylation (bottom panel) (n = 5 per group). * *P *< 0.05 versus control, nonventilated mice; **†***P *< 0.05 versus all other groups. Akt^+/- ^= Akt deficient mice; LMWH = enoxaparin; O_2 _= mice with hyperoxia; RA = mice with room air.

We measured the activity of Akt in lung homogenate of mice exposed to V_T _30 ml/kg mechanical ventilation for 2 to 8 hours while breathing room air or hyperoxia to determine the effects of hyperoxia on stretch-induced Akt phosphorylation. There were time-dependent increases in the phosphorylation of Akt but there were no significant changes in the expression of total nonphosphorylated proteins of Akt. The activation of Akt increased after 2 hours of mechanical ventilation with V_T _30 ml/kg and remained increased after 8 hours of mechanical ventilation compared with those of control, nonventilated mice (Figure 6). To further determine the inflammatory cells involved in the hyperoxia-induced Akt activation, we measured Akt activity in isolated neutrophils from lung tissue (Figure 7). The increases in Akt activation in mice receiving V_T _30 ml/kg mechanical ventilation with hyperoxia were significantly reduced after pharmacological inhibition with enoxaparin (Figure 5B and 7).

### Akt deficient mice reduced the effects of hyperoxia on ventilation-induced microvascular leak, Akt activation, HMGB1 mRNA expression, neutrophil sequestration, oxygen radicals, and HMGB1 production

**Figure 8:**
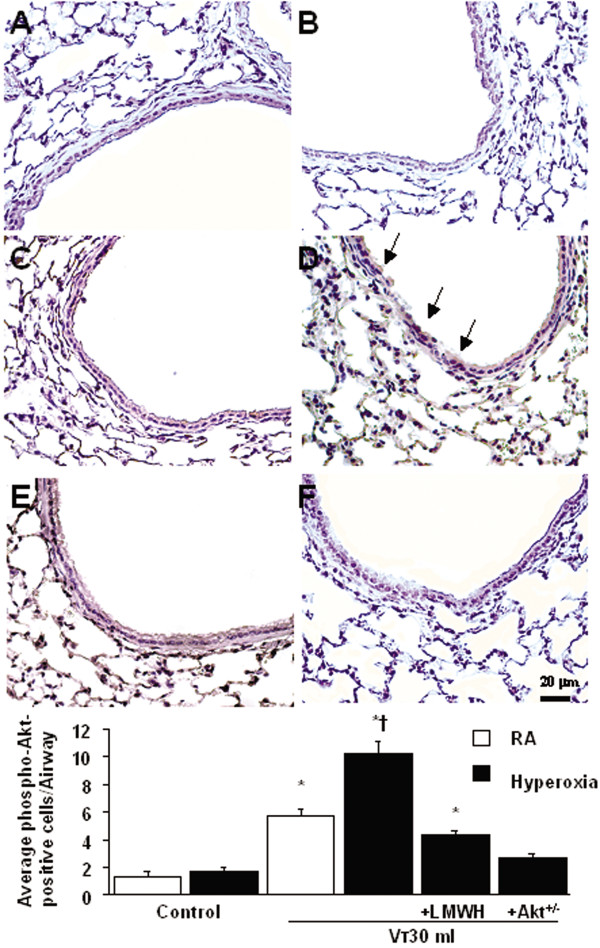
**Enoxaparin and Akt deficient mice reduced hyperoxia-augmented lung stretch-induced Akt activation in airway epithelium**. Representative photomicrographs (x400) with phospho-Akt staining of paraffin lung sections with immunohistochemistry were from control, nonventilated mice and mice ventilated at V_T _30 ml/kg for 8 hours with room air or hyperoxia. Enoxaparin, 4 mg/kg, was given subcutaneously 30 minutes before ventilation (n = 5 per group). (A) Control wild-type mice with room air; (B) Control wild-type mice with hyperoxia; (C) V_T _30 ml/kg wild-type mice with room air; (D) V_T _30 ml/kg wild-type mice with hyperoxia; (E) V_T _30 ml/kg wild-type mice pretreated with 4 mg/kg enoxaparin breathing hyperoxia; (F) V_T _30 ml/kg Akt deficient mice with hyperoxia. A dark brown diaminobenzidine (DAB) signal identified by arrows indicates positive staining for phospho-Akt in the lung epithelium or interstitial, whereas shades of bluish tan signify nonreactive cells. * *P *< 0.05 versus control, nonventilated mice; **†***P *< 0.05 versus all other groups. Scale bars represent 20 μm. Akt^+/- ^= Akt deficient mice; LMWH = enoxaparin; RA = mice with room air.

To determine the role of Akt activation in hyperoxia-augmented VILI, we used Akt deficient mice. The effects of hyperoxia on the increases of microvascular leak, lung edema, MPO levels, HMGB1 mRNA expression, Akt activation in whole lung homogenate and isolated neutrophils from lung tissue, and HMGB1 production in mice receiving V_T _30 ml/kg mechanical ventilation were significantly reduced with Akt deficient mice (Figure 1, 3, 5, and 7). Reduced production of MDA but increased production of total GSH was found in Akt deficient mice (Figure 2). The reduction between V_T _30 ml/kg mechanical ventilation using hyperoxia with and without Akt deficient mice was more than those between V_T _30 ml/kg mechanical ventilation with and without hyperoxia. This suggested that Akt deficient mice reduced both effects of mechanical ventilation and hyperoxia. With immunohistochemistry, we further confirmed the effectiveness of enoxaparin in the inhibition of HMGB1 production and activation of Akt signaling involved in hyperoxia-augmented high V_T_-induced lung inflammation in bronchial epithelial cells (Figure 8).

## Discussion

Mechanical ventilation with hyperoxia, both in the early and late fibroproliferative stages of ALI, is often required to prevent hypoxemia in patients with ARDS. Prolonged exposure to hyperoxia may lead to excessive production of reactive oxygen species, which are generated by neutrophils, macrophages, and airway epithelial cells [21]. In a previous *in vivo *mouse study of ALI, we found that hyperoxia increased high V_T_-induced lung inflammation and production of MIP-2 chemoattractant, a functional homolog of human interleukin (IL)-8 belonging to the cysteine-amino-cysteine family of cytokines [6]. On exposure to hyperoxia, excessive reactive oxygen species act as direct cell toxins and as secondary messengers by inducing pulmonary epithelium to secrete chemoattractants and proinflammatory cytokines that lead to influx of neutrophils to the lung [21]. In the present mouse model of VILI, we found that hyperoxia increased high V_T_-induced lung edema, microvascular permeability, neutrophil infiltration, and free radical production. High tidal volumes in normal animals have been used to mimic overdistention of the less injured and thus more compliant areas of lung found in ARDS patients. We explored further the roles of neutrophils and Akt activation in hyperoxia-augmented lung injury and production of HMGB1, a lethal mediator of severe sepsis.

Hyperoxia-induced lung injury is characterized by overwhelming diffuse inflammation, pulmonary edema, destruction of alveolar-capillary barrier, and loss of lung compliance [5]. In a previous study of rats, we found the addition of oxygen to the rats ventilated at V_T _20 ml/kg markedly increased the bronchoalveolar fluid content of neutrophils [2]. Neutrophils are the major inflammatory cells involved in the process of acute lung injury and play a major role in the generation of reactive oxygen species [8]. In the present study, we isolated neutrophils from lung tissues and found mechanical ventilation with hyperoxia significantly increased the activation of Akt signaling.

HMGB1 has been shown to be a late onset biomarker of lethal systemic inflammation, which distinguishes it from the early acting mediators of IL-8, IL-1β, and TNF-α [9]. There are three HMGB nonhistone chromosomal proteins: HMGB1 (previously HMG1), HMGB2 (previously HMG2), and HMGB3 (previously HMG4 or HMG2). HMGBs are composed of three different domains, including homologous DNA binding boxes A and B in the N-terminus and the C-terminal domain [22]. HMGB1 appears to have two distinct functions in cellular systems, as an intracellular regulator of transcription and an extracellular inflammatory cytokine [22]. A previous study of ALI in mice pretreated with HMGB1 showed that lung inflammation with neutrophil accumulation and local production of proinflammatory cytokines, including IL-1β, TNF-α, and MIP-2 [23]. We found that hyperoxia increased mechanical ventilation-induced influx of neutrophils and HMGB1 production. Reduced levels of glutathione have been found in the alveolar fluid of patients with ARDS[8]. In our study, high-tidal-volume mechanical ventilation with hyperoxia increased the levels of malondialdehyde, an aldehydic secondary product of lipid peroxidation used as a marker of oxidative damage and decreased the levels of glutathione, the most abundant non-protein thiol functioning as an endogenous scavenger of reactive oxygen species in the presence of glutathione peroxidase by reducing hydrogen peroxide or other hydroperoxides to less toxic substances [24,25]. In a study of injurious mechanical ventilation in rabbits, bronchoalveolar lavage fluid levels of HMGB1 were 5-fold higher after 4 hours of mechanical ventilation with large tidal volumes compared with those of lower tidal volumes [10]. We found that hyperoxia with production of free radicals and recruitment of neutrophils augmented high V_T_-induced HMGB1 mRNA expression and HMGB1 production.

Heparin was shown to modulate infiltration of neutrophils and improve gas exchange and severity of hyaline membrane formation [26,27]. An *in vitro *study of endothelia demonstrated that HMGB1 interacted in a complex manner with the hemostatic system and PAI-1 was important for HMGB1 to exert its effects [28]. Our previous murine study of ALI showed that enoxaparin reduced high-tidal-volume ventilation-induced PAI-1 production, an early mediator of acute pulmonary inflammation [16]. In the present study, we demonstrated further that hyperoxia increased high tidal volume ventilation-induced HMGB1 mRNA expression and HMGB1 production, a late mediator of lung inflammation, in a short duration of mechanical ventilation. Pharmacological inhibition with enoxaparin reduced the levels of HMGB1 involved in ALI. Previous study of high-tidal-volume mechanical ventilation using hyperoxia in mice showed that Akt signaling in lung epithelial cells regulated neutrophil migration into the injured lungs [29]. In an *in vitro *study of human neutrophils, others showed that HMGB1 increased nuclear translocation of nuclear factor-κappa-B and expression of proinflammatory cytokines among neutrophils through activation of p38, extracellular signal-regulated kinase 1/2, and PI 3-K/Akt [22,30]. In our murine study, we found hyperoxia augmented high V_T_-induced neutrophil sequestration, free radical production, HMGB1 mRNA expression, Akt activation in bronchial epithelium and neutrophils, and HMGB1 production. These effects were significantly reduced with Akt deficient mice. HMGB1^-/- ^mice are not used in this study because they die shortly after birth due to a defect in the transcriptional activation of the glucocorticoid receptor, consistent with the notion that HMGB1 functions as a regulator of transcription involving steroid hormone receptors [31].

Findings in other study supported our results that neutrophil infiltration and the development of acute lung injury involved the Akt pathway through toll-like receptors (TLR) 4-dependent activation of nicotinamide adenine dinucleotide phosphate oxidase and reactive oxygen species production in a mouse model of hemorrhagic shock and resuscitation [32]. HMGB1-mediated neutrophil activation occurs through TLR2, TLR4, and advanced glycation end products while LPS-induced signals through TLR4 [33]. The heterogeneity of peptides that interact with HMGB1 indicates that HMGB1 does not have a highly preferred interaction sequence and is capable of associating with multiple proteins [34]. Our study does have limitation that we don't compare the differences between different sexes [35]. Incorporation of hormone variations in further experiments may explore more about whether male and female mice are equally susceptible to VILI.

## Conclusion

Though the ARDS network trial demonstrated that low-tidal-volume ventilation is safer than high-tidal-volume ventilation, these findings have been questioned and the mechanisms of injury and protection need to be further examined [36]. The National Heart, Lung and Blood Institute working group on acute lung injury identified examination of the biology of stress-induced injury to the lung in health and disease as a fertile area of future research, because ventilation-induced release of cytokines may lead to systemic translocation and multisystem organ failure [37]. High tidal volumes in normal animals have been used to mimic overdistention of the less injured and thus more compliant areas of lung found in ARDS patients. These animal models have shown that simply over overdistending lung tissue, in the absence of acid aspiration or bacterial lipopolysaccharide causes production of cytokines and chemokines [3,38,39]. In clinical practice after intubations, patients are often ventilated initially with high levels of tidal volume and oxygen (fraction of inspired oxygen of more than or equal to 50%) to oxygenate the brain and other vital organs. Using an *in vivo *mouse model, we have found that hyperoxia increased high-tidal-volume ventilation-induced epithelial cell injury, resulted in increased pulmonary neutrophil sequestration, oxidant stress, and increased HMGB1 production. The deleterious effects were attenuated by low-dose enoxaparin treatment and were, at least in part, dependent on the Akt pathway. Our data suggest that effects of high-tidal-volume ventilation and hyperoxia on lung inflammation occur in the first few hours of VILI. Understanding the protective mechanism of low-dose enoxaparin related with the regulation of HMGB1 and Akt signaling may advance the growing knowledge of biomarkers to be monitored in the course of ALI and the effects of mechanical forces in the lung involved in the pathogenesis of biotrauma with hyperoxia.

## List of abbreviations

Akt: serine/threonine kinase/protein kinase B; ALI: acute lung injury; ARDS: acute respiratory distress syndrome; BAL: bronchoalveolar fluid; DAB: diaminobenzidine; EBD: Evans blue dye; ERK 1/2: extracellular signal regulated kinases 1/2; GAPDH: glyceraldehydes-phosphate dehydrogenase; GSH: total glutathione; HMGB1: high-mobility group box-1; IHC: immunohistochemical stain; IL: interleukin; JNK: c-Jun NH_2_-terminal kinase; LMWH: low molecular weight heparin; LPS: lipopolysaccharide; MDA: malondialdehyde; MIP-2: murine macrophage inflammatory protein-2; MPO: myeloperoxidase; NF-κB: nuclear factor-κB; PAI-1: plasminogen activator inhibitor-1; PI3-K: phosphoinositide 3-OH kinase; RT-PCR: reverse transcription-polymerase chain reaction; TLR: toll-like receptors; TNF-α: tumor necrosis factor-α; VILI: ventilator-induced lung injury; V_T_: tidal volume.

## Competing interests

The authors declare that they have no competing interests.

## Authors' contributions

L-LF collected and analyzed the data. Y-CT, H-CC, L-YY, K-KC, and L-HC reviewed and coordinated the study.

All authors have read and approved the final manuscript.
